# Supplementary material for: A general strategy for the ultrafast surface modification of metals
Source: Nat Commun. 2016 Dec 7;7:13797. doi: 10.1038/ncomms13797 (PMC5151094; doi:10.1038/ncomms13797)
Supplement: Supplementary Information — Supplementary Figures 1-4 [file ncomms13797-s1.pdf]

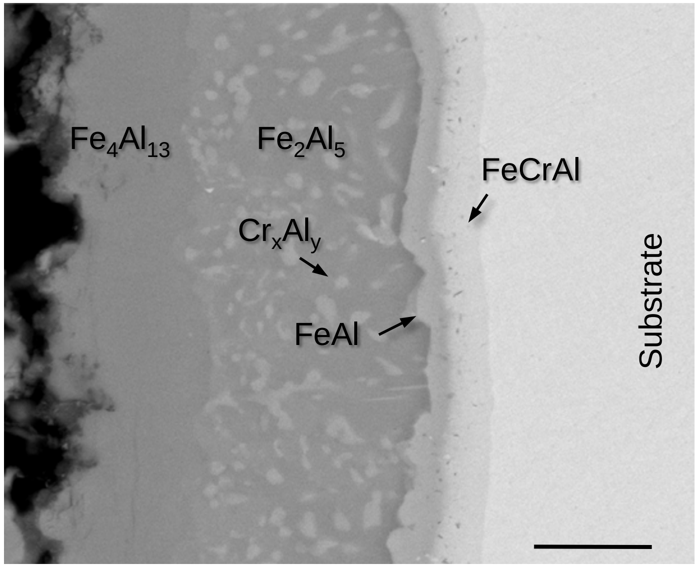

Supplementary Figure 1 | Conventional aluminizing. SEM image of the cross-section of the samples aluminized in furnace at 860 °C for 10 minutes, indicating that the microstructure of the aluminized layers is similar to that of the DC-aluminized sample. Scale bar, 10 µm.

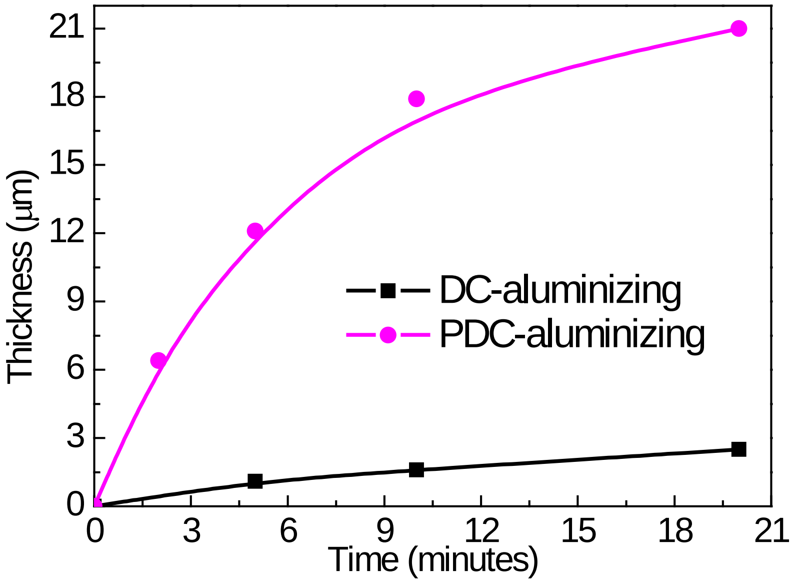

Supplementary Figure 2 | Growth kinetics of β-FeAl layer. The growth kinetic curves for the growth of β-FeAl layer in DC- and PDC-aluminized samples.

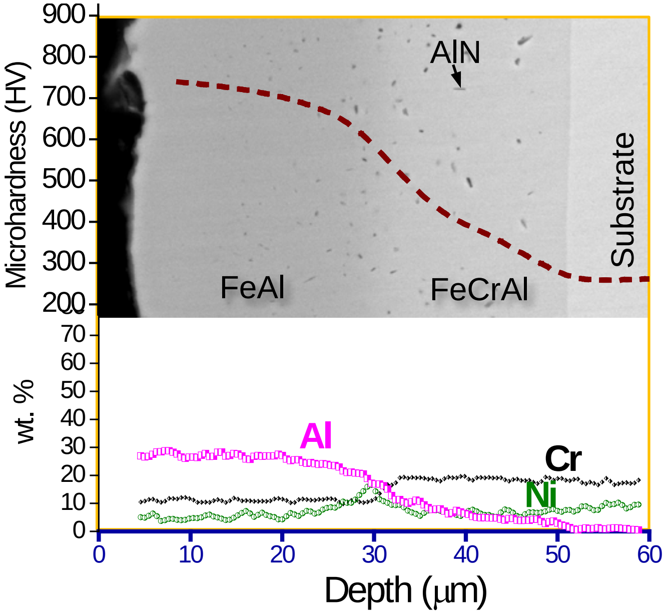

Supplementary Figure 3 | Characterization of the AC-aluminized layer. SEM image, EDS composition and indentation depth profile of the cross-section of the aluminized layer after passing **A**C at 1200 A cm^{-2} for 10 minutes.

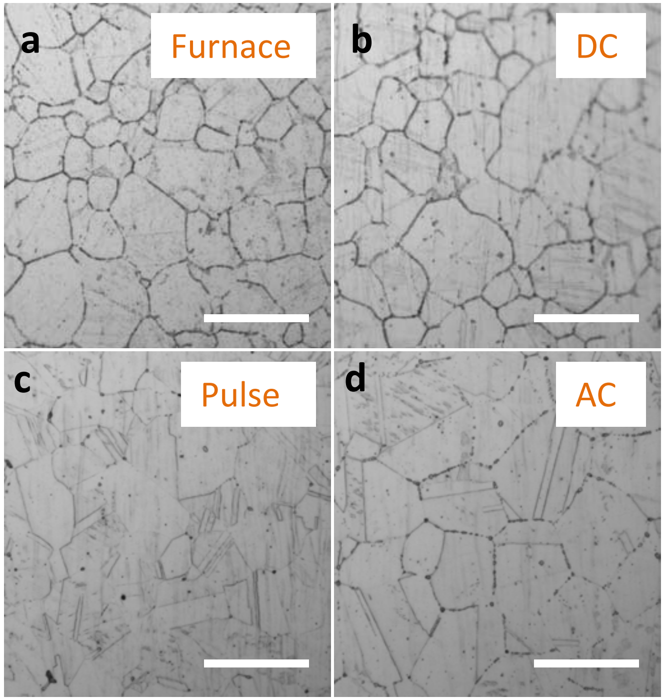

Supplementary Figure 4 | Metallographic examinations of the substrate after aluminizing. a, Furnace aluminizing (860 ^{o}C, 10 minutes), mean grain size: 18.3 μm, number of twin boundaries per unit length (twin density): 0.05 $μm^{-1}$. **b**, DC-aluminizing (1300 A cm^{-2}, 10 minutes), mean grain size: 19.4 μm, twin density: 0.07 $μm^{-1}$. **c**, PDC-aluminizing (1400 A cm^{-2}, 10 minutes), mean grain size: 19.4 μm, twin density: 0.09 $μm^{-1}$. **d**, AC-aluminizing (1200 A cm^{-2}, 10 minutes), mean grain size: 22.2 μm, twin density: 0.06 $μm^{-1}$. Scale bar, 50 μm.
